# Risk Factors for Excessive Gestational Weight Gain in a Healthy, Nulliparous Cohort

**DOI:** 10.1155/2014/148391

**Published:** 2014-06-03

**Authors:** Antonia Restall, Rennae S. Taylor, John M. D. Thompson, Deralie Flower, Gustaaf A. Dekker, Louise C. Kenny, Lucilla Poston, Lesley M. E. McCowan

**Affiliations:** ^1^Department of Obstetrics and Gynaecology, Faculty of Medical and Health Sciences, University of Auckland, Private Bag 92019, Auckland 1142, New Zealand; ^2^Department of Paediatrics, Faculty of Medical and Health Sciences, University of Auckland, Private Bag 92019, Auckland 1142, New Zealand; ^3^Women and Children's Division, Lyell McEwin Hospital, University of Adelaide, Adelaide, SA 5112, Australia; ^4^The Irish Centre for Fetal and Neonatal Translational Research (INFANT), Department of Obstetrics and Gynaecology, University College Cork, Ireland; ^5^Department of Maternal and Fetal Medicine and the Division of Women's Health, Women's Health Academic Centre, King's College London and King's Health Partners, London SE1 7EH, UK; ^6^South Auckland Clinical School and Auckland City Hospital, Auckland 1142, New Zealand

## Abstract

*Objective*. Excessive gestational weight gain (GWG) is associated with adverse maternal and child outcomes and contributes to obesity in women. Our aim was to identify early pregnancy factors associated with excessive GWG, in a contemporary nulliparous cohort. *Methods*. Participants in the SCOPE study were classified into GWG categories (“not excessive” versus “excessive”) based on pregravid body mass index (BMI) using 2009 Institute of Medicine (IOM) guidelines. Maternal characteristics and pregnancy risk factors at 14–16 weeks were compared between categories and multivariable analysis controlled for confounding factors. *Results*. Of 1950 women, 17% gained weight within the recommended range, 74% had excessive and 9% inadequate GWG. Women with excessive GWG were more likely to be overweight (adjOR 2.9 (95% CI 2.2–3.8)) or obese (adjOR 2.5 (95% CI 1.8–3.5)) before pregnancy compared to women with a normal BMI. Other factors independently associated with excessive GWG included recruitment in Ireland, younger maternal age, increasing maternal birthweight, cessation of smoking by 14–16 weeks, increased nightly sleep duration, high seafood diet, recent immigrant, limiting behaviour, and decreasing exercise by 14–16 weeks. Fertility treatment was protective. *Conclusions*. Identification of potentially modifiable risk factors for excessive GWG provides opportunities for intervention studies to improve pregnancy outcome and prevent maternal obesity.

## 1. Introduction

The Institute of Medicine (IOM) guidelines for pregnancy weight gain were initially developed in 1990 to promote adequate gestational weight gain (GWG) with the goal of preventing premature births and small-for-gestational-age infants [1]. However, with increasing numbers of women entering pregnancy overweight and obese, the IOM guidelines for pregnancy weight gain were recently updated in 2009 with a shift of focus towards maternal health outcomes and reduction of postpartum weight retention and childhood adiposity [2]. The updated 2009 IOM guidelines for GWG utilise standard body mass index (BMI) categories developed by the World Health Organisation and provide a relatively narrow GWG target for obese women in pregnancy (Table 1) [2].

The increasing prevalence of overweight and obesity has created a global epidemic with associated substantial personal and health care costs. Excessive GWG increases the likelihood of postpartum weight retention and long-term weight gain and is an important contributor to the obesity epidemic among women [3, 4].

Women who gain weight in excess of the 2009 IOM recommendations also have an increased risk of adverse maternal and child outcomes [2]. Specifically, excessive GWG has been associated with an increased risk of hypertensive disorders of pregnancy [5, 6], large for gestational age (LGA) infants [6–10], and nonelective Caesarean delivery [6, 9–11]. Excessive GWG has also been associated with the development of childhood adiposity and obesity, thereby contributing to obesity in both mothers and their offspring [12–15]. Mechanistically, excessive GWG may expose the developing fetus to persistently raised concentrations of glucose, insulin, amino acids, and lipids as well as inflammatory cytokines derived from maternal adipose tissue [16]. During periods of developmental plasticity, it is hypothesised that fetal pathways of energy balance may be permanently “reset” by this adverse environment, leading to a metabolic predisposition to obesity [15, 17]. Alternatively, or in addition, heritable predisposition to gain weight or common lifestyle factors which promote weight gain (e.g., low levels of physical activity, high energy diet) may contribute to the shared risk of obesity between mother and child [16].

In previous reports, risk of excessive GWG has been variously associated with the mother's prepregnancy BMI and dietary and lifestyle behaviours [18–32]. Parity has seldom been considered, although risk factors may differ between nulliparous and parous women due to differences in lifestyle and family environment. Since more than 40% of births in western countries typically occur in nulliparous women [33–35], we aimed to identify early pregnancy risk factors for excessive GWG in nullipara. This study was undertaken using data on GWG in women recruited to the screening for pregnancy endpoints (SCOPE) cohort, a large international study of healthy women in their first pregnancies. We recently reported that more than 70% of women in this study had excessive GWG according to the recent IOM guidelines [36]. We proposed that identification of risk factors might allow the development of targeted strategies and interventions to help women at the greatest risk achieve optimal weight gain during their pregnancy and lower their risk of obstetric complications and future obesity for both themselves and their infants.

## 2. Materials and Methods

### 2.1. Study Design and Ethics Approval

The screening for pregnancy endpoints (SCOPE) study is a prospective, multicentre cohort study with primary aims of developing screening tests to identify women at risk of preeclampsia, small-for-gestational-age (SGA) infants, and spontaneous preterm birth and is registered with the Australia New Zealand Clinical Trial Registry: ACTRN12607000551493. The participants comprised healthy nulliparous women with singleton pregnancies. Full details of the study design and methods have been published elsewhere [37, 38]. Participants in this component of the SCOPE study were recruited between November 2004 and February 2011, in Adelaide, Australia, Auckland, New Zealand, and Cork, Ireland. The three United Kingdom SCOPE centres (Manchester, Leeds, and London) were not included in this study of GWG due to the fact that end of pregnancy weights were not being routinely recorded. Ethical approval was obtained from local ethics committees (Australia REC 1712/5/2008, New Zealand AKX/02/00/364, and Cork ECM5 (10) 05/02/08) and all participants provided written informed consent.

**Figure 1:**
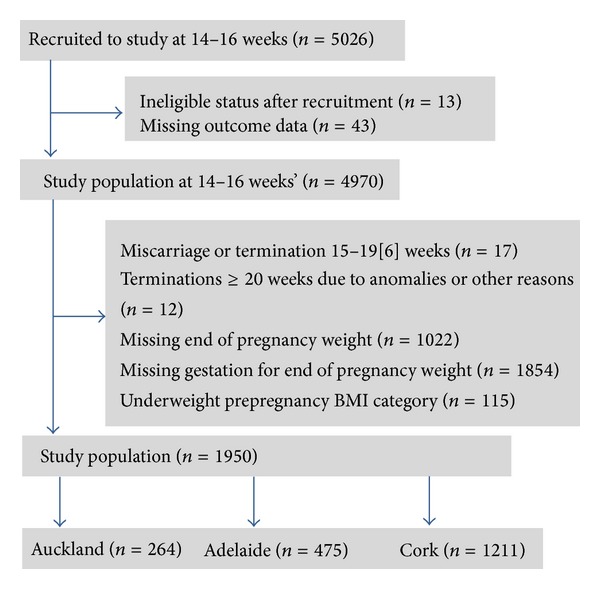
Recruitment flow chart.

All participants were seen at 14–16 weeks of gestation at which time they completed an extensive interview. Physical measurements including height and weight were measured by a research midwife during this visit. The data were then entered into an internet accessed central database with a complete audit trail (MedSciNet^AB^, Stockholm, Sweden). Participants were followed prospectively and data on pregnancy outcome were collected by a research midwife, usually within 72 hours of birth. Women included in this study comprised those who had weight recorded at 14–16 weeks of gestation and at the end of pregnancy. The total GWG for the second and third trimesters were calculated and categories of weight gain were assigned according to 2009 IOM guidelines [2]. In order to estimate as accurately as possible prepregnancy BMI, 1.25 kg (the average of 0.5–2.0 kg weight gain in the first trimester reported in the IOM 2009 guideline) was subtracted from each participant's weight at 14–16 weeks of gestation [2, 18]. Prepregnancy BMI was then categorised according to WHO criteria (underweight BMI <18.5 kg/m^2^, normal BMI 18.5–24.9 kg/m^2^, overweight BMI 25.0–29.9 kg/m^2^, and obese BMI ≥ 30.0 kg/m^2^). Exclusion criteria in this study are outlined in Figure 1. Women who were underweight were excluded due to small numbers. Each participant's weight gain per week in the second and third trimester was adjusted for the gestation at delivery and calculated by the following formula:* GWG (kg/week) = total weight gain (kg)/(week at final weight measurement − week at first visit measurement)* [18, 39]. For 49% of the total SCOPE cohort, the gestational week at which the final weight measurement was recorded was not recorded. These participants were excluded from the current study (Figure 1). Participants were classified as gaining “less than,” “within,” or “exceeding” the recommended rates of weight gain based on their prepregnancy BMI group [2]. The groups of women who gained below or within the recommended ranges (“not excessive GWG”) were then compared to those who exceeded the ranges for GWG (“excessive GWG”).

### 2.2. Definitions

The estimated date of delivery was calculated from a certain last menstrual period (LMP) date and was only adjusted if either (1) a scan performed at <16 weeks of gestation found a difference of ≥7 days between the scan gestation and that calculated by the LMP or (2) on 20-week scan a difference of ≥10 days was found between the scan gestation and that calculated from the LMP. If the LMP date was uncertain, then scan dates were used to calculate the estimated date of delivery. Socioeconomic index, a measure of socioeconomic status (higher score indicating a higher status), was based on the New Zealand socioeconomic index [40]. Fertility treatment was defined as any treatment given to assist conception of the current pregnancy, including hormonal treatment, artificial insemination,* in vitro* fertilisation, and intra-cytoplasmic sperm injection. High fish or seafood intake was defined as ≥3 servings of any fish (including oily fish such as tuna or salmon) or seafood (including shellfish or shrimps) per week. The limiting behaviour score is adapted from the “Behavioural Responses to Illness Questionnaire” and assesses the extent to which a participant has limited their normal activities and exercise since becoming pregnant [41]. A higher limiting score reflects greater limitation of activities. Pregnancy-induced hypertension included either gestational hypertension defined as sBP ≥ 140 mmHg and/or dBP ≥ 90 mmHg on at least 2 occasions 4 hours apart after 20 weeks of gestation but before the onset of labour or preeclampsia defined as gestational hypertension plus proteinuria ≥300 mg/24 h or spot urine protein : creatinine ratio ≥30 mg/mmol creatinine or urine dipstick protein ≥ ++ or any multisystem disease [42]. SGA and LGA were defined as birth weight less than the 10th and greater than the 90th customised centile, respectively, and were adjusted for maternal height, booking weight, and ethnicity as well as gestational age at delivery and sex of the infant [43].

### 2.3. Statistical Analysis

All analyses were performed using the statistical software package SPSS 19 (SPSS Inc., version 19.0, Chicago, IL, USA). Categorical and continuous variables were analysed by Chi-square tests and Student's* t*-test, respectively, to compare maternal demographic and lifestyle factors with “excessive” versus “not excessive” gestational weight gain categories. Statistical significance was defined at the 5% level.

In total, univariable analyses were performed on 300 variables of interest based on* a priori* knowledge using binary logistic regression modelling to assess the risk of excessive gestational weight gain. Variables were then excluded due to a* P* value >0.1 (205 variables), variables with >10% missing data (20 variables), and variables with a cell count <5 (10 variables). Of the remaining 65 variables, 21 were selected for multivariable modelling based on known risk factors or variables of interest. The initial variable list used to create the multivariable model is available as a supporting file in Supplementary Table 1A in the Supplementary Material available online at http://dx.doi.org/10.1155/2014/148391 and the remaining 44 variables deleted are in a supporting file in Supplementary Table 1B. Of the selected variables, data were complete in >99% of participants for each variable except participants' birth weight (4.15% missing data). Data for missing variables were imputed using the mode for categorical data variables (*n* = 2) and expectation maximization (*n* = 1) or median (*n* = 2) for continuous data variables. Multivariable analysis using a backward stepwise method controlling for each variable in the model was performed.

## 3. Results

5026 nulliparous women with singleton pregnancies were recruited into the SCOPE study from the three participating centres. Of these women, 1950 were eligible for the current study, 475 from Adelaide, 264 from Auckland, and 1211 from Cork (Figure 1). The demographics of those who were included compared to those who were excluded for the three participating SCOPE centres are shown in Table 2. There were no significant differences in either Auckland or Cork, whilst those who participated in the current study from Adelaide were slightly older (24.3 versus 23.4 years), had a slightly higher BMI (27.5 versus 26.7 kg/m^2^), and were less likely to smoke (20 versus 27%). The main reasons for exclusion were missing data for end of pregnancy weight or gestation at which the end of pregnancy weight was recorded. The average weight gain from 14–16 weeks of gestation until the last weight measured in pregnancy was 12.31 ± 5.26 kg across the cohort and was normally distributed. The estimated total weight gain [2] for this study cohort from conception until end of pregnancy was 13.55 kg, 13.91 ± 4.62 kg for normal, 13.76 ± 5.19 kg for overweight, and 11.82 ± 7.18 kg for obese women. Only 335 women (17.2%) gained weight within the ranges recommended by the 2009 IOM guidelines and 166 (8.5%) of participants failed to gain sufficient weight during pregnancy. Accordingly, 1449 (74.3%) women in our study had excessive GWG. These women were more likely to be overweight or obese compared to women who gained within or below 2009 IOM guidelines (Table 3). They were also at greater risk of Caesarean section in labour, LGA infants, and hypertensive disorders of pregnancy.

After identification of statistically significant variables associated with excessive GWG in univariable analyses, a multivariable logistic regression model was developed to estimate factors independently associated with excessive GWG. The unadjusted and adjusted OR and corresponding 95% confidence intervals are shown in Table 4.

In the final multivariable model, overweight women at 14–16 weeks' gestation were nearly three times as likely to exceed GWG ranges (95% CI 2.20, 3.82) compared to those with a normal BMI, while obese women were approximately 2.5 times more at risk of excessive GWG (95% CI 1.79, 3.52). Compared to the referent group of women aged 35 years or more, younger women had a higher risk of excessive GWG, with women aged less than 25 years at an almost 2-fold increase in risk. Additional models controlling for maternal educational status and family income level (as a marker of socioeconomic status) were run. These had no effect on the relationship between maternal age and excessive GWG in the model. Women who ceased smoking in pregnancy also had elevated risk (adjusted OR 1.67, 95% CI 1.17, 2.35). Additionally, the risk of excessive GWG increased by 15% (CI 1.02–1.28) for every 500 g increase in maternal birth weight. No relationship between maternal birth weight and infant birth weight was found (*R* ^2^ = 0.037).

Hours of sleep on weekday nights were also positively correlated with excessive GWG with those women who slept for 10 or more hours a night being at higher risk of excessive GWG by nearly twofold compared to women who reported sleeping less than 8 hours. Women who reported a decrease in exercise by 14–16 weeks were 50% more likely to have excessive GWG and women who recorded greater limiting behaviours were more likely to gain above the 2009 IOM guidelines (adjusted OR = 1.04, CI 1.01–1.07). Other factors associated with excessive GWG included a high intake of fish or seafood (adjusted OR = 2.75, CI 1.45, 5.22), being a migrant in the previous five years (adjusted OR = 1.57, CI 1.05, 32.35), living in Cork (adjusted OR = 2.31, CI 1.63, 3.29) compared to Auckland. The use of fertility treatment to conceive the current pregnancy was the only protective factor identified (adjusted OR 0.59, CI 0.37–0.93). The relationship between fertility treatment and infant birth weight was not significant (*P* = 0.98).

## 4. Discussion

In this cohort of healthy, nulliparous pregnant women recruited between 2004 and 2011, nearly three quarters (74.3%), gained weight in excess of the 2009 IOM guidelines. Total weight gain was approximately two kilograms less in obese participants but because of the lower recommended optimal GWG in obese women they were 2.5 times as likely (CI 1.77–3.51) to exceed IOM weight gain guidelines. Similarly overweight women were also at increased risk of excessive GWG. Our findings are consistent with previous reports which indicate that high pregravid/early pregnancy BMI is a strong predictor of excessive GWG, despite absolute weight gains being lower compared to women with a normal BMI [19–22, 27–32]. These findings are concerning as excessive GWG is an important pathway to postpartum weight retention and the development of new or persistent obesity in women of reproductive age and also their offspring [15, 44]. This has major implications for long-term individual health and future health care costs to a society already burdened by obesity.

Our study has shown that excessive GWG is influenced by nonmodifiable risk factors including maternal age and birth weight. Women aged less than 25 years and 25–29 years are almost twice as likely and women 30–34 years 60% are more likely to gain above IOM guidelines compared to women aged 35 years or more. Our findings regarding age are consistent with the majority of previous reports which have found that young mothers are at greater risk of exceeding GWG guidelines [26, 28, 30] whereas older women tend to gain less weight during pregnancy [18, 28, 30]. It is unusual for advanced maternal age to be a protective factor against an adverse outcome. Possible explanations include a poor anabolic response to pregnancy or alternatively older women may be more disciplined regarding lifestyle choices. We also report a novel independent association between the mother's birth weight and her risk of excessive GWG (aOR 1.17, CI 1.06–1.29 per 500 g) which was not explained by a correlation between maternal and infant birth weight. A possible explanation is that higher birth weight women are themselves programmed for positive energy balance. In contrast, the use of fertility treatment to conceive the current pregnancy was associated with an approximate 40% lower risk of excessive GWG, which was not explained by lower infant birth weight.

Women who quit smoking before 14–16 weeks of gestation were 50% more likely to exceed 2009 IOM GWG guidelines compared to nonsmokers. This was not accounted for by a difference in birth weight between nonsmokers and ceased smokers which were virtually identical. Weight gain after stopping smoking is likely multifactorial. Withdrawal of the appetite suppressant effects of nicotine and replacement of smoking with consumption of foods high in sugar and fat which activate neural reward pathways similar to those activated by smoking may all contribute to the overconsumption of energy on quitting smoking [45]. Only limited previous evidence is available regarding smoking cessation during pregnancy and risk of excessive GWG [23, 25, 28]. Favaretto et al. found that quitting smoking after conception increased the odds of excessive GWG by 34% (95% CI 1.10–1.63) compared to nonsmokers [23]. Interestingly, Favaretto et al. alsonoted that women who quit smoking within six months of conception gained on average 1.0 kg more than nonsmokers, compared to an extra 1.5 kg gained by women who quit smoking after conception [23]. It may therefore be advisable to counsel women on the benefits of quitting prior to conception in an attempt to limit excessive GWG, as well as providing comprehensive nutritional and exercise advice to those who quit in pregnancy.

We also examined a number of lifestyle factors which may impact on GWG. While excess television viewing (>5 hours per day) and computer usage were associated with excessive GWG in our univariable analyses, these factors were no longer significant in the multivariable model. Sleep duration on weekday nights remained an independent risk factor for excessive GWG in our multivariable analysis. Women who slept for ten hours or more were nearly twice as likely to exceed 2009 IOM recommendations compared to women who reported less than eight hours of sleep nightly. While these results are contrary to findings from Althuizen et al., which links a reduction in nocturnal sleep with increased GWG [19], a larger study has recently identified sleep deprivation as a risk factor for inadequate GWG [46]. When we compared sleep duration in the “low” versus “normal” GWG groups we found no significant difference. The mechanism underlying our findings is not understood but may include less time available for physical activity. Further research is needed to fully elucidate the influence of sleep on weight gain in pregnancy. Our findings also show, as might be anticipated, that women who exhibit greater “limiting behavior” [41] (e.g., by restricting their usual level of activity, avoiding physical exercise, going to bed during the daytime, and putting their life on hold) are at greater risk of excessive GWG.

Dietary intake was also assessed during the 14–16 weeks of gestation SCOPE visit. Although reported intake of fruits and vegetables did not influence GWG, consuming three or more servings of fish or seafood per week was an independent risk factor for excessive GWG (adjOR = 2.96, CI 1.53, 5.74). Further exploration of our data confirmed that this relationship between high fish intake and excessive gestational weight gain was not a surrogate for eating deep fried hot potato chips (“fish and chips”) as the association between high fish intake and excessive GWG remained after adjustment for chips. It is possible that high fish or seafood intake may reflect a participant's total energy intake, a factor which has previously been associated with excessive GWG but was not measured in our cohort [19, 24, 27, 30]. An effect of fish on satiety may also be possible as a recent small randomised controlled trial of fish oil supplements reported that supplementation was associated with increased appetite in female participants [47].

It is unclear why the cohort of women in Cork had a higher risk of excessive GWG. It is possible that local societal-cultural factors regarding expected weight gain in pregnancy (i.e., eating for two) may have played a part.

Strengths of our study include the novel study population comprising a cohort of healthy, nulliparous women, and the large subgroup with excessive GWG [20–22, 24, 30, 32]. In addition, all anthropometric measurements were obtained by a research midwife to ensure that BMI at 14–16 weeks of gestation was calculated accurately, thereby eliminating the potential for self-reporting bias. Our findings are also based on the recently updated 2009 IOM pregnancy weight gain guidelines [2] and are adjusted for gestational length rather than total weight gain.

Several factors which may limit the reliability of our study however should be noted. Prepregnancy weight measurements were not available and have been estimated using methodology recommended by the 2009 IOM guidelines [2]. However, this will have minimal impact on the value of prepregnancy BMI as weight gain in the first trimester of pregnancy is small. Additionally, it is possible that modifiable behaviours assessed at 14–16 weeks of gestation did not reflect behaviours over the course of the pregnancy.

## 5. Conclusion

Nearly three quarters of the participants in our cohort had excessive GWG. We have identified some modifiable and nonmodifiable risk factors for excessive GWG. Women at increased risk could be targeted for future intervention studies.

## Supplementary Material

Supporting File Information: Sixty-five variables were potentially available for the gestational weight gain model. The initial variable list used to create the multivariable model is available in the supporting file in Supplementary Table 1A and the remaining 44 variables deleted are in a supporting file in Supplementary Table 1B.

## Figures and Tables

**Table 1:** Institute of Medicine guidelines for recommended gestational weight gain based on prepregnancy body mass index [5].

Body mass index	Gestational weight gain (kg/wk)		
	Low	Normal	Excessive
Normal weight (18.5–24.9 kg/m^2^)	<0.35	0.35–0.50	>0.50
Overweight (25.0–29.9 kg/m^2^)	<0.23	0.23–0.33	>0.33
Obese (≥30.0 kg/m^2^)	<0.17	0.17–0.27	>0.27

**Table 2:** Demographic details by centre of those included and excluded from study.

	Auckland cohort			Adelaide cohort			Cork cohort		
	Included	Excluded	*P *	Included	Excluded	*P *	Included	Excluded	*P *
	*N* = 264	*N* = 1752		*N* = 475	*N* = 679		*N* = 1211	*N* = 560	
Age (years)*	30.4 (4.9)	30.4 (4.7)	0.88	24.3 (5.0)	23.4 (5.2)	0.005	29.9 (4.5)	30.1 (4.5)	0.39
Caucasian ethnicity*	216 (82)	1480 (85)	0.27	435 (92)	624 (92)	0.85	1183 (98)	547 (98)	0.99
Body mass index (kg/m^2^)*	24.5 (3.7)	24.8 (4.2)	0.36	27.5 (6.2)	26.7 (6.8)	0.04	25.0 (4.2)	24.6 (4.1)	0.07
Socioeconomic index score*	48 (15)	48 (15)	0.61	28 (10)	28 (10)	0.59	42 (16)	43 (16)	0.06
Smoking status*			0.72			0.008			0.39
Nonsmoker	230 (87)	1545 (88)		313 (66)	389 (57)		872 (72)	411 (73)	
Stopped during pregnancy	25 (10)	141 (8)		68 (14)	108 (16)		210 (17)	101 (18)	
Current smoker	9 (3)	66 (4)		94 (20)	182 (27)		129 (11)	48 (9)	

Data are mean (SD) or number (%) as appropriate.

*Data collected at 14–16 weeks of gestation.

*P* value comparison is between “included in cohort” versus “not included in cohort” GWG groups using Chi-square or independent samples *t*-test.

**Table 3:** Maternal characteristics and pregnancy outcomes by gestational weight gain categories (*n* = 1950).

	Weight gain category (by 2009 IOM guidelines)		
	Not excessive (*n* = 501; 25.7%)	Excessive (*n* = 1449; 74.3%)	*P*
Recruitment centre			<0.001
Adelaide	157 (31.3)	318 (22.0)	
Cork	243 (48.5)	968 (66.8)	
Auckland	101 (20.2)	163 (11.2)	
Age (years)*	28.7 (5.6)	28.5 (5.2)	0.67
Ethnicity			0.10
Caucasian	468 (93.4)	1366 (94.3)	
Asian	12 (2.4)	15 (1.0)	
Polynesian (Maori & Pacific Island)	3 (0.6)	18 (1.2)	
Indian	10 (2.0)	20 (1.4)	
Other (including African)	8 (1.6)	30 (2.1)	
Socioeconomic index*	39.5 (16.0)	39.4 (16.1)	0.97
Body mass index (kg/m^2^)*	24.6 (4.8)	25.9 (4.8)	<0.001
Smoking status*			0.001
Nonsmoker	380 (75.8)	1035 (71.4)	
Stopped during pregnancy	53 (10.6)	250 (17.3)	
Current smoker	68 (13.6)	164 (11.3)	
Pregnancy-induced hypertension	54 (10.8)	242 (16.7)	0.001
Preeclampsia	16 (3.2)	79 (5.5)	
Gestational hypertension	38 (7.6)	163 (11.2)	
Mode of delivery			0.001
Caesarean section (prelabour)	35 (7.0)	154 (10.6)	
Caesarean section (in labour)	77 (15.4)	295 (20.4)	
Vaginal birth	389 (77.6)	1000 (69.0)	
Birth weight (g)	3301 (522)	3523 (535)	<0.001
Gestation at delivery (wks)	39.8 (1.8)	39.9 (1.6)	0.12
SGA infant	77 (15.4)	135 (9.3)	<0.001
LGA infant	21 (4.2)	198 (13.7)	<0.001

Data are mean (SD) or number (%) as appropriate.

IOM: Institute of Medicine; SGA: small for gestational age; LGA: large for gestational age.

*Data collected at 14–16 weeks' of gestation.

*P* value comparison is between “not excessive” versus “excessive” GWG groups using Chi-square or independent samples *t*-test.

**Table 4:** Multivariable associations with excessive gestational weight gain (*n* = 1950).

	Weight gain category (by 2009 IOM guidelines)			
	Not excessive (*n* = 501; 25.7%)	Excessive (*n* = 1449; 74.3%)		
	*n* (%) or mean ± SD	*n* (%) or mean ± SD	Unadjusted OR (95% CI)	Adjusted OR (95% CI)^†^
Centre				
Adelaide	157 (31.3)	318 (22.0)	1.26 (0.91–1.72)	1.03 (0.69–1.53)
Cork	243 (48.5)	968 (66.8)	2.47 (1.84, 3.32)	2.31 (1.63–3.29)
Auckland	101 (20.2)	163 (11.2)	1.0	1.0
Age (years)*				
≤24	126 (25.1)	321 (22.2)	1.24 (0.86, 1.78)	1.92 (1.24–2.97)
25–29	134 (26.8)	457 (31.5)	1.66 (1.17, 2.36)	1.88 (1.29–2.73)
30–34	167 (33.3)	519 (35.8)	1.51 (1.08, 2.13)	1.59 (1.11–2.27)
≥35	74 (14.8)	152 (10.5)	1.0	1.0
BMI groups (kg/m^2^)*				
18.5–24.9	350 (69.9)	724 (50.0)	1.0	1.0
25.0–29.9	90 (17.9)	477 (32.9)	2.56 (1.98–3.32)	2.90 (2.20–3.82)
≥30.0	61 (12.2)	248 (17.1)	1.97 (1.45–2.67)	2.50 (1.79–3.52)
Smoking status*				
Nonsmoker	380 (75.8)	1035 (71.4)	1.0	1.0
Stopped during pregnancy	53 (10.6)	250 (17.3)	1.73 (1.26–2.38)	1.67 (1.18–2.36)
Current smoker	68 (13.6)	164 (11.3)	0.89 (0.65–1.20)	0.94 (0.65–1.36)
Mother's birth weight per 500 g increase^†^	3228 (585)	3353 (546)	1.22 (1.11–1.34)	1.15 (1.02–1.28)
Immigrant in past 5 years				
No	460 (26.3)	1290 (20.5)	1.0	1.0
Yes	41 (73.7)	159 (79.5)	1.38 (0.95, 2.01)	1.57 (1.05–2.35)
Fertility treatment				
No	465 (92.8)	1381 (95.3)	1.0	1.0
Yes	36 (7.2)	68 (4.7)	0.64 (0.41, 0.99)	0.59 (0.37–0.93)
Fish or seafood intake (servings/week)*				
<3	489 (97.6)	1365 (94.2)	1.0	1.0
≥3	12 (2.4)	84 (5.8)	2.51 (1.36–4.63)	2.75 (1.45–5.22)
Limiting behaviour score^‡^	7.2 (3.7)	8.0 (4.0)	1.05 (1.03–1.08)	1.04 (1.01–1.07)
Exercise in pregnancy*				
Unchanged	200 (39.9)	452 (31.2)	1.00	1.0
Increased	27 (5.4)	71 (4.9)	1.16 (0.71, 1.92)	0.91 (0.55–1.51)
Decreased	274 (54.7)	926 (63.9)	1.50 (1.20, 1.86)	1.30 (1.01–1.69)
Sleep (hours/weeknight)*				
<8	143 (28.5)	323 (22.3)	1.0	1.0
8-9	302 (60.3)	904 (62.4)	1.33 (1.05–1.68)	1.40 (1.09–1.81)
≥10	56 (11.2)	222 (15.3)	1.76 (1.23–2.50)	1.83 (1.24–2.69)

Data are mean (SD) or number (%) as appropriate.

CI: confidence interval; BMI: body mass index; IOM: Institute of Medicine; OR: odds ratio; SD: standard deviation.

*Data collected at 14–16 weeks of gestation.

^†^Multivariate regression model presented as odds ratios with 95% confidence intervals. Multivariable model controls for all variables in the table.

^‡^The limiting behaviour score ranges from 0 to 20 with higher values corresponding with greater limiting behaviour; OR refers to a one unit change in score.
