# Supplementary material for: Risk Factors for Excessive Gestational Weight Gain in a Healthy, Nulliparous Cohort
Source: J Obes. 2014 Jun 3;2014:148391. doi: 10.1155/2014/148391 (PMC4065732; doi:10.1155/2014/148391)
Supplement: Supplementary file 1 — Supporting File Information: Sixty-five variables were potentially available for the gestational weight gain model. The initial variable list used to create the multivariable model is available in the supporting file in Supplementary Table 1A and the remaining 44 variables deleted are in a supporting file in Supplementary Table 1B. [file 148391.f1.pdf]

Supplementary Table 1A. Variables submitted to the model with *P* value <0.10

Variable Name	Variable Type	Comments
Centre	1 Adelaide<br>2 Cork<br>3 Auckland	*P*=<0.001
Body mass index groups (kg/m^2)	1 18.5 - 24.9<br>2 25.0 - 29.9<br>3 ≥30	Data collected at 14-16 week SCOPE visit.<br>*P*=<0.001
Age group (years)	1 <25<br>2 25 - 29<br>3 30 - 34<br>4 ≥35	Data collected at 14-16 week SCOPE visit.<br>*P*=0.013
Immigrant status	Yes<br>No	Participant migration in the previous five years.<br>*P*=0.076
Education (years)	1 <12<br>2 12 - 13<br>3 >13	Data collected at 14-16 week SCOPE visit.<br>*P*=<0.001
Participant born preterm	Yes<br>No	Participant born preterm (<37 weeks). Data was self-reported but women were asked to bring their 'birth record' if kept at home.<br>*P*=0.003; 0.92%; missing, imputed.
Fertility treatment	Yes<br>No	Fertility treatment to conceive current pregnancy. Includes *in vitro* fertilisation, intra-cytoplasmic sperm injection, clomiphene or other hormonal treatment, donor oocyte, donor

		sperm or artificial insemination.<br>*P*= 0.032
Fish or seafood intake (servings per week)	1 <3<br>2 ≥3	Includes servings of oily fish, other fish and seafood prior to 14 - 16 week SCOPE visit.<br>*P*=0.002
Folate at first visit	Yes<br>No	Any folate at 14 - 16 week SCOPE visit.<br>*P*=0.001
Multivitamin in first trimester	Yes<br>No	Any multivitamin intake in 1st trimester.<br>*P*=0.024
Smoking status	1 non-smoker<br>2 stopped during pregnancy<br>3 current smoker	Data collected at 14-16 week SCOPE visit.<br>*P*=0.001
Recreational drug use in first trimester	Yes<br>No	Consumed/inhaled/injected recreational drugs in the first trimester.<br>*P*=0.044
Binge alcohol in first trimester	Yes<br>No	Any binge alcohol in the first trimester.<br>*P*=0.003
Exercise in pregnancy	1 decreased<br>2 unchanged<br>3 increased	Change in exercise level in pregnancy. Data collected at 14-16 week SCOPE visit.<br>*P*=0.001; 0.31% missing, imputed.
Excess television viewing	Yes<br>No	Watching ≥5 hours/day. Data collected at 14-16 week SCOPE visit.<br>*P*=0.079

Computer use in pregnancy	Yes<br>No	Any computer usage in past month. Data collected at 14-16 week SCOPE visit.<br>*P*=0.063
Sleep duration on weekday nights (hours)	1 <8<br>2 8-9<br>3 ≥10	How many hours of sleeping at night on weekdays on average at 14 - 16 week SCOPE visit.<br>*P*=0.004; 0.31% missing, imputed
Mother's birthweight (per 500g increase)	Continuous	Data was self-reported but women were asked to bring their 'birth record' if kept at home.<br>*P*=<0.001; 4.15% missing, imputed
Limiting behaviour score	Continuous	The Limiting Behaviour score ranges from 0 to 20 with higher values corresponding with greater limiting behaviour.<br>*P*=<0.001; 0.36% missing, imputed.
High leafy vegetable intake	1 <3 serves/day<br>2 ≥3 serves/day	High (>=3 times per day) green leafy vegetables consumption in pregnancy prior to 14 -16 week SCOPE visit.<br>*P*=0.025
Deep fried hot potato chip intake	Yes<br>No	Any deep fried hot potato chip intake prior to 14 - 16 week SCOPE visit.<br>*P*=0.005; 0.15% missing, not imputed.

Supplementary Table 1B. Variables removed from the model with *P* value < 0.10

Variable Name	Variable Type	Comments
*Pre-pregnancy body mass index (kg/m^2)	Continuous	Data collected at 14-16 week SCOPE visit and estimated according to IOM guidelines.<br>*P*=<0.001
*Pre-pregnancy body mass index (kg/m^2)<br>(3 group categories)	1 18.5 - 24.9<br>2 25.0 - 29.9<br>3 ≥30	Data collected at 14-16 week SCOPE visit.<br>*P* =<0.001
*Body mass index (kg/m^2)	Continuous	Data collected at 14-16 week SCOPE visit.<br>*P* =<0.001
*Pre-pregnancy body mass index (kg/m^2)<br>(4 group categories)	1 <18.5 underweight<br>2 ≥18.5 and <25 normal weight<br>3 ≥25 and <30 overweight<br>4 ≥30 obese	Data collected at 14-16 week SCOPE visit and categorized into 4 groups according to World Health Organization criteria. .<br>*P* =<0.001
*Weight (kg)	Continuous	Data collected at 14-16 week SCOPE visit.<br>*P* =<0.001
*Estimated pre-pregnancy weight (kg)	Continuous	Data collected at 14-16 week SCOPE visit and estimated according to IOM guidelines.<br>*P* =<0.001
*Height (cm)	Continuous	Data collected at 14-16 week SCOPE visit.<br>*P* =<0.001
*Height < 161 cm	Yes	Short stature (< 161 cm).

	No	*P* =<0.001
* ⸸Waist circumference (cm)	Continuous	Data collected at 14-16 week SCOPE visit<br><br>*P* =<0.001
* ⸸Waist ≥ 94 cm	Yes<br>No	Large waist ≥ 94 cm. Data collected at 14-16 week SCOPE visit.<br><br>*P* =0.025
*⸸Waist to height ratio	Continuous	Data collected at 14-16 week SCOPE visit.<br><br>*P* =0.005
*Arm circumference (cm)	Continuous	Mid upper arm circumference at 14-16 week SCOPE visit.<br><br>*P* =<0.001
*Participant birthweight group<br><br>(4 group categories)	1 <1500g<br>2 1500-2499g<br>3 2500-3499g<br>4 >-3500g	Participant's birthweight divided into categories.<br><br>*P* =<0.001
*In vitro fertilization	Yes<br>No	In vitro fertilization to conceive in current pregnancy.<br><br>*P* =0.007
*Intracytoplasmic sperm injection	Yes<br>No	Intracytoplasmic sperm injection to conceive in current pregnancy.<br><br>*P* =0.021
*In vitro fertilization or intracytoplasmic sperm injection	Yes<br>No	Either in vitro fertilization or intracytoplasmic sperm injection to conceive in current pregnancy.<br><br>*P* =0.019
*Oily fish	1 Often (≥3x per	Frequency consumed oily fish

consumption<br><br>(2 group categories)	week)<br>2 Moderate (1-2x per week) or Rarely	(which is high in omega 3 long chain fatty acids) in pregnancy prior to 15w SCOPE visit, 2 severity grades. Deleted as overlapped with similar variable<br><br>*P* =0.007
*Oily fish consumption<br><br>(3 group categories)	1 Often (≥3x per week)<br>2 Moderate (1-2x per week)<br>3 Rarely (≤3x per month)	Frequency consumed oily fish (which is high in omega 3 long chain fatty acids) in pregnancy prior to 15w SCOPE visit, 3 severity grades. Deleted as overlapped with similar variable<br><br>*P* =0.030
*High oily fish consumption	Yes<br><br>No	High consumption oily fish (which is high in omega 3 long chain fatty acids) ≥3 times a week in pregnancy prior to 15w SCOPE visit.<br><br>*P* =0.009
*Other fish consumption pre-pregnancy<br><br>(8 group categories)	1 Never<br>2 1-3/mth<br>3 1 or 2/wk<br>4 3 or 4/wk<br>5 5 or 6/wk<br>6 1-2/day<br>7 3-4/day<br>8 ≥5/day	Frequency consumed other fish (excludes oily fish) or seafood in the month prior to conception.<br><br>*P* =0.072
*High other fish consumption pre-pregnancy	Yes<br><br>No	Consumption of other fish (excludes oily fish) or seafood<br><br>≥3 times a week in the month prior to conception.<br><br>*P* =0.085
*Green leafy	1 Never	Frequency consumed green

vegetables pre-pregnancy<br><br>(8 group categories)	2 1-3/mth<br>3 1 or 2/wk<br>4 3 or 4/wk<br>5 5 or 6/wk<br>6 1-2/day<br>7 3-4/day<br>8 ≥5/day	leafy vegetables in the month prior to conception.<br><br>*P* =0.079
*High folate intake in 1st trimester	1 Not taking folate<br>2 Yes <=800<br>3 Yes >800	Folate dose by <=800µg and >800µg per day in 1st trimester.<br><br>*P* =0.084
*High folate intake at 14-16 weeks visit<br><br>(3 group categories)	1 Not taking folate<br>2 Yes ≤800<br>3 Yes >800	Folate dose by <=800µg and >800µg per day at 15w SCOPE visit.<br><br>*P* =0.001
*Multivitamin intake in 1st trimester<br><br>(3 group categories)	1 No<br>2 Less than daily<br>3 Daily	Frequency of multivitamin intake in 1st trimester.<br><br>*P* =0.015
*Any multivitamin intake at 14-16 weeks visit	Yes<br><br>No	Any multivitamin intake at 15w SCOPE visit.<br><br>*P* =0.041
*Smoking status at 14-16 week visit<br><br>(4 group categories)	1 Never smoked<br>2 Smoked pre-preg, but quit smoking before pregnant<br>3 Smoked in preg, but quit smoking before 1st visit<br>4 Smoking at 1st visit	Smoking status at 15w SCOPE visit in 4 groups (pre-pregnancy and pregnancy smoking status).<br><br>*P* =0.004
*Cigarettes per day 1st trimester	Continuous	Number of cigarettes per day in the 1st trimester.<br><br>*P* =0.097
*Cigarette exposure prior to 14-16 week	1 No smoking<br>2 1-12wk	Number of weeks of cigarette exposure in pregnancy prior to

visit<br><br>(3 group categories)	3 >12wk	15w SCOPE visit (categories).<br><br>P=0.005
*Cigarettes per day at 14-16 week visit	Continuous	Number of cigarettes per day at 14-16 week SCOPE visit.<br><br>*P* =0.064
*Smoked in 1st trimester	Yes<br>No	Smoked during the 1st trimester.<br><br>P=0.056
*Alcohol intake pre-pregnancy<br><br>(5 group categories)	1 No alcohol<br>2 1-2<br>3 3-7<br>4 8-14<br>5 >14	Units of alcohol per week in the 3 months pre-pregnancy (5 categories).<br><br>*P* =0.004
*Alcohol intake pre-pregnancy<br><br>(3 group categories)	1 No Alcohol<br>2 Low Alcohol consumption (≤2units/day or ≥14units/week)<br>3 High Alcohol consumption (>2 units/day or >14 units/wk)	Units of alcohol per week in the 3 months pre-pregnancy divided into 3 grades of severity.<br><br>*P* =0.052
*Alcohol intake 1st trimester<br><br>(5 group categories)	0 No alcohol<br>1 1-2<br>2 3-7<br>3 8-14<br>4 >14	Units of alcohol per week in the 1st trimester (5 categories).<br><br>*P* =0.065
*Any recreational drug use in 1st trimester	Yes<br>No	Consumed/inhaled/injected other recreational drugs in the 1st trimester combining 'unknown' (n=0) with No.<br><br>*P* =0.042
*Gestation ceased recreational drug use	1 No other drugs taken<br>2 1-12 weeks	Gestation ceased other recreational drugs

(3 group categories)	3 >12 weeks	(categories).<br>P=0.019
*Any marijuana use 1st trimester	Yes<br>No	Used any marijuana in the 1st trimester.<br>*P* =0.098
*Any marijuana use at 14-16 week visit	Yes<br>No	Any use of marijuana during pregnancy at 14-16 week visit.<br>*P* =0.098
*Exercise/gardening at14-16 week visit	Continuous	Hours exercising/gardening per week evaluated at 14-16 week visit.<br>*P* =0.018
*Exercise/gardening at 14-16 week visit<br>(7 group categories)	0 No exercise/gardening<br>1 1 hour<br>2 2 hours<br>3 3 hours<br>4 4 hours<br>5 5 hours<br>6 6≥ hours	Hours exercising/gardening per week evaluated at 14-16 week visit by 7 categories.<br>*P*=0.009
*Climbing stairs per day<br>(3 group categories)	1 Never<br>2 <10x/day<br>3 ≥10x/day	Number of times climbed stairs in the last month, evaluated at 15w SCOPE visit.<br>*P* =0.028
*Limiting behaviour >90th centile	Yes<br>No	Behavioural response to pregnancy: Limiting Behaviour Score evaluated at 15w SCOPE visit >90th Centile.<br>*P* =0.003
*All or None Behaviour Score	Continuous	Behavioural response to pregnancy: All or None Behaviour Score evaluated at 15w SCOPE visit: All or None

		Behaviour Score.<br>*P* =0.049
*Sleep duration on weekday nights (hours)	Continuous	How many hours of sleeping at night on weekdays on average at 14-16 week SCOPE visit.<br>*P* =0.002

* Deleted as overlapped with similar variable already in the model.

† Deleted as difficult to replicate in future clinical practice.
